# Depression in multiple system atrophy: Views on pathological, clinical and imaging aspects

**DOI:** 10.3389/fpsyt.2022.980371

**Published:** 2022-09-08

**Authors:** Qiuyi Lv, Yuxin Pan, Xing Chen, Jingpei Wei, Wei Wang, Hua Zhang, Jifeng Wan, Shiqiang Li, Yan Zhuang, Baolin Yang, Dayong Ma, Dawei Ren, Zijun Zhao

**Affiliations:** ^1^Department of Neurology and Stroke Center, Dongzhimen Hospital, The First Affiliated Hospital of Beijing University of Chinese Medicine, Beijing, China; ^2^Institute of Neuroscience, Chinese Academy of Sciences, Shanghai, China

**Keywords:** multiple system atrophy, depression, pathology, clinical feature, imaging

## Abstract

Multiple system atrophy (MSA) is a common atypical parkinsonism, characterized by a varying combination of autonomic, cerebellar, and pyramidal systems. It has been noticed that the patients with MSA can be accompanied by some neuropsychiatric disorders, in particular depression. However, there is limited understanding of MSA-related depression. To bridge existing gaps, we summarized research progress on this topic and provided a new perspective regarding pathological, clinical, and imaging aspects. Firstly, we synthesized corresponding studies in order to investigate the relationship between depression and MSA from a pathological perspective. And then, from a clinical perspective, we focused on the prevalence of depression in MS patients and the comparison with other populations. Furthermore, the associations between depression and some clinical characteristics, such as life quality and gender, have been reported. The available neuroimaging studies were too sparse to draw conclusions about the radiological aspect of depression in MSA patients but we still described them in the presence of paper. Finally, we discussed some limitations and shortcomings existing in the included studies, which call for more high-quality basic research and clinical research in this field.

## Introduction

Multiple system atrophy (MSA) is a rare, sporadic progressive neurodegenerative disease, characterized clinically by a variable combination of parkinsonism, cerebellar dysfunction, pyramidal signs, and autonomic dysfunction (1). The prevalence of MSA is between 3.4 and 4.9 cases per 100,000 people, making MSA an orphan disease (2). The average age of onset is 54–63 years, men are more often affected, and there is a 6–11- year survival rate (3). MSA severely reduces the life quality and expectancy of patients and has resulted in an enormous economic and public health burden (4, 5). The main features include autonomic, pyramidal, extrapyramidal, and cerebellar categories in any combination. Many MSA patients present with orthostatic hypotension (OH) and genitourinary dysfunction due to autonomic neuropathy, and then go on to develop balance, speech, and coordination dysfunction within a few years (6, 7). The two principal subtypes are distinguished by their initial predominant extrapyramidal motor deficits: the parkinsonian subtype (MSA-P), also called Striatonigral degeneration (SND), presents as Parkinsonism predominantly, including bradykinesia, muscle rigidity, tremors, and postural instability; the cerebellar subtype (MSA-C), also called Olivopontocerebellar atrophy (OPCA), exhibits prominent upper motor neuron and cerebellar signs related to pontine and olivocerebellar degeneration (8). It has been reported that MSA-P is more prevalent in Europe and North America, whereas MSA-C is more prevalent in Japan (9, 10). However, whether environmental and genetic factors confer susceptibility to the MSA has remained controversial until recently. As a common type of parkinsonian syndrome, MSA and Parkinson's disease (PD) have some overlapping clinical manifestations but have some pathophysiological distinctions. It has been suggested that the abnormal deposition of protein α-synuclein (α-Syn) may play a critical role in the initiation and progression of PD and MSA (11). However, in patients with PD, α-Syn predominantly aggregates in neurons forming Lewy bodies and Lewy neurites; while in patients with MSA, α-Syn aggregates mainly in oligodendroglial cells forming glial cytoplasmic inclusions (GCIs) (7).

Besides motor and autonomic symptoms, patients affected by MSA often complain of neuropsychiatric disorders, including depression, anxiety, emotional lability and pseudobulbar affect, which result in a worsening of quality of life (QoL) and an increase in disability (12–15). Considering that not many studies have investigated neuropsychiatric disorders in MSA patients, we only focus on the occurrence of depression in this review. Depression is a common and frequently severe neuropsychiatric disturbance, affecting 350 million people worldwide (16). An understanding of the factors associated with depression is important to social care. It's generally accepted that depressive symptoms are more common in neurodegenerative diseases and other chronic disease groups compared to the general population (15). Previous studies have shown remission of depressive symptoms to be associated with improved functional recovery (17). Correspondingly, evidence notes that depression is a strong predictor of physical illness and early death in neurological diseases (18). However, it is not known whether depression is a part of the disease spectrum or is secondary to the severity of motor or autonomic impairment in MSA patients (19, 20). It is also unclear whether the occurrence of depression in MSA has its own particularity when compared to other neurological diseases. To answer these questions, we reviewed the literature available on depressive symptoms in patients with MSA. We performed focused literature searches using PubMed and Google Scholar from 1980 to January 2022 to identify relevant original research and review articles. Search terms focused on neuropathology (e.g., “depression” “MSA” “oxidative stress,” inflammation”), associations between depression symptoms and MSA (e.g., “depression,” “MSA,” “prevalence”), and neuroimaging of depression in MSA (e.g., “depression,” “MSA,” “positron emission tomography,” “magnetic resonance imaging,” “diffusion tensor imaging,” “neuroimaging”). Titles and abstracts from these studies were obtained, full-text articles were screened for relevant manuscripts, and reference lists were reviewed to supplement additional manuscripts appropriate for review. For the purposes of this paper, we first summarized the relationship between depression and MSA from a pathological perspective. And then, we discussed the clinical and radiological aspects of depression in MSA. Furthermore, the main evaluation scales validated for depression in MSA were reported.

## Neuropathology of depression in MSA

**Figure 1:**
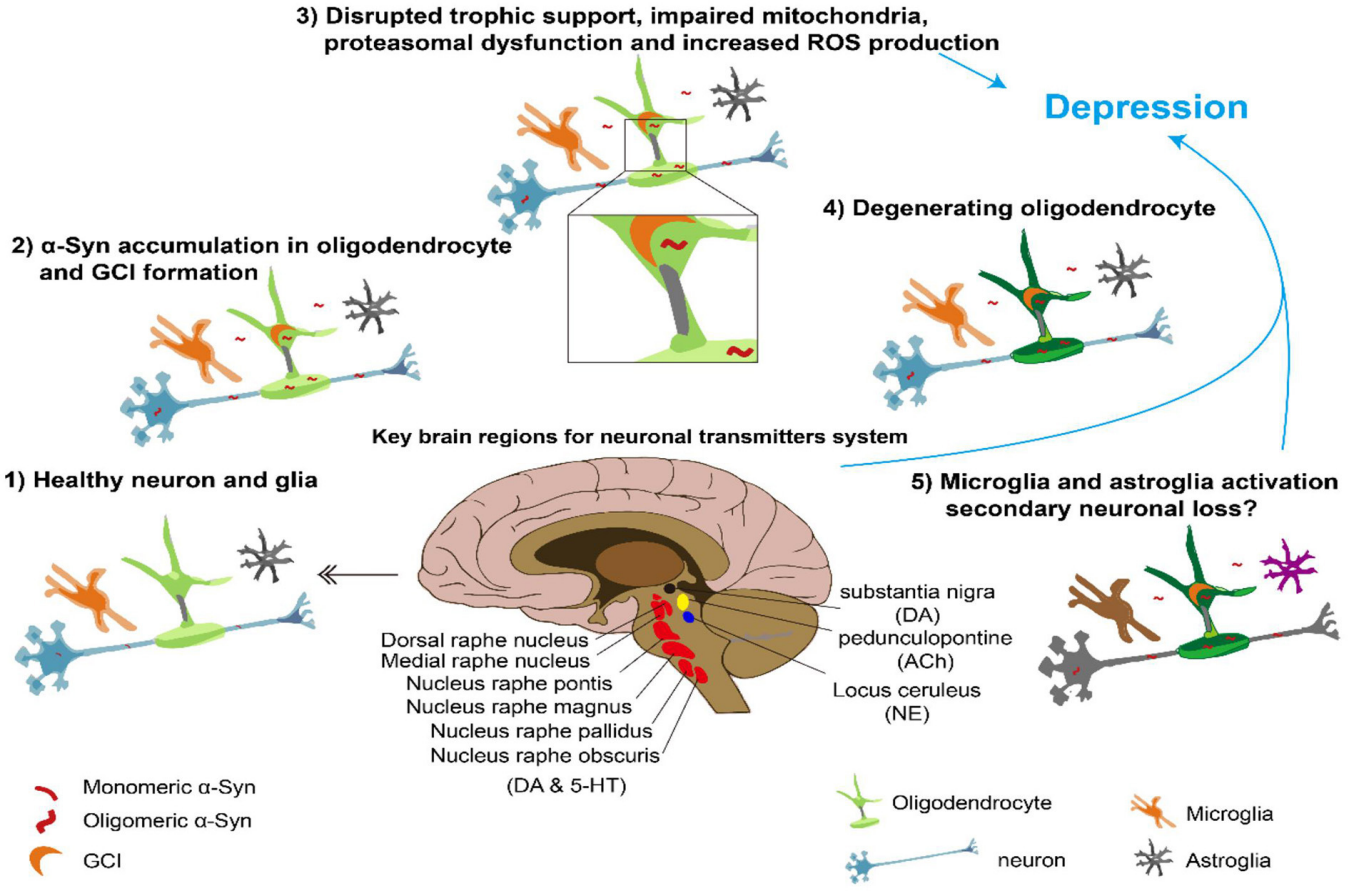
Overview of pathological a-synuclein accumulation leading to MSA and depression Center, brain regions important for neural transmitter involved in depression. Surround, possible process underlying MSA and depression. DA, dopamine; ACh, acetyl choline; NE, norepinephrine; 5- HT, serotonin.

Current knowledge of the pathological mechanisms underlying MSA places great emphasis on the α-synuclein (α-Syn) accumulation in the oligodendrocyte, which leads to the formation of insoluble half-moon-shaped GCIs that are characteristic of the disease. GCIs further cause disrupted trophic support, impaired mitochondria, proteasomal dysfunction, and increased reactive oxygen species (ROS) production, which leads to oligodendrocyte degeneration. Finally, microglial and astrocyte-glial activation may cause secondary neuronal loss (21). Some of the cells that die during this process are those secreting important neural transmitters or those located in important brain regions. Since the two subtypes of MSA have different pathologies with cell loss in distinct brain regions, their relationships with depression may also be different. MSA-P is characterized by more severe cell loss in the substantia nigra, putamen, and globus pallidus, whereas MSA-C is characterized by more cell loss in the inferior olives, pontine nuclei, and Purkinje cells (21, 22). A summary of the potential pathology underlying MSA and depression is depicted in Figure 1.

### Neural transmitter abnormalities

The neural transmitter system, such as dopamine (DA), norepinephrine (NE), serotonin (5-HT), and acetylcholine, has been the focus of most studies into the mechanisms underlying depression (23, 24). Since it was proposed, the “monoamine hypothesis” has dominated for more than half a century, which attributes depression to the abnormal function of monoamine systems (25–27). Depressive symptoms were thought to be caused by a lack of central noradrenergic or serotonergic systems, whereas mania was thought to be caused by a functional excess of monoamines.

In an autopsy study, the authors used immunohistochemistry to identify serotonergic neurons in the rostral raphe nuclei and found relative preservation of serotonergic neurons in four patients with MSA compared to healthy controls (28). However, subsequent studies with a larger sample size found that although dorsal raphe (DR) and median raphe (MR) nuclei in rostral raphe nuclei preserve serotonergic cell bodies, caudal raphe (medullary raphe nuclei) experiences consistent neuronal loss (29). Screening of more medullary regions revealed more brain areas susceptible to serotonergic neuronal loss, including the nucleus raphe magnus, nucleus raphe obscurus, nucleus raphe pallidus, and ventrolateral medulla in MSA patients (30). Depletion of medullary serotonergic neurons, on the other hand, has been linked to the sudden death of MSA patients *via* a potential contribution of these neurons to autonomic failures such as cardiovascular and respiratory system regulation (31). Evidence from transgenic mouse models of MSA supports this idea. The raphe obscurus and pallidus of mice expressing human wild-type -synuclein under the control of a proteolipid promoter (PLP-SYN) show less serotonergic immunoreactivity (32).

The decrease in nigrostriatal dopamine activities in PD patients, as well as the association between dopamine and the reward system, has prompted research into the relationship between depression and PD (23). Similar logic applies to MSA, especially for the MSA-P type. Further evidence from PLP-α-synuclein transgenic mice also suggested dopaminergic neuronal loss in the substantia nigra pars compacta (SNc) between the ages of 2 and 4 months, in parallel to the microglial activation that was thought to be the typical pathology of MSA. Furthermore, these changes could be reversed by inhibiting microglial activation with minocycline (33, 34).

Based on the results of patients' autopsy, these LC neurons that serve as a major source of norepinephrine were found to be depleted in MSA patients than in healthy controls (28, 33). Evidence was consistently based on neuroimaging results that visualize neuromelanin-related contrast of the noradrenergic neurons in LC, where the contrast ratio of LC was most prominently decreased in MSA patients (35). Further research showed a marked loss of nerve cells in LC for both MSA-P and MSA-C subtypes (36). Studies have also shown a cholinergic neuronal loss in pedunculopontine in MSA patients (28, 37). Because the dominance of cholinergic neurotransmitter activities over adrenergic neurotransmitter activities was thought to play a role in depression, cholinergic cell loss in MSA may also contribute to depression manifestation (38, 39). Taken together, the neural transmitter dysfunction mentioned above may be a common denominator between MSA and depression pathology.

### Inflammation and oxidative stress

Aside from the “monoamine hypothesis,” inflammatory mechanisms also play a role in the cause of depression (40). Maes et al. proposed that increased monocytic production of interleukins (including IL-1β and IL-6) is the key phenomenon underlying immune response and contributing to hypothalamic-pituitary-adrenal axis hyperactivity, serotonin metabolism disorder, and vegetative symptoms in severe depression (41). Clinical observations support this idea from several angles. Inflammatory markers and proinflammatory cytokines are increased in MDD, while reduction of inflammatory biomarkers is correlated with antidepressant treatment. Moreover, depressed patients with higher inflammation biomarker levels are more likely to develop a treatment resistance (42).

One important source of inflammation is oxidative stress, and they are closely correlated with each other in pathophysiological processes, one of which can be easily induced by another (43). In brief, inflammation causes reactive oxygen species (ROS) production and antioxidant depletion, which further induces a reduction in levels of antioxidants and causes oxidative stress. In turn, increased oxidative stress causes an increase in the expression of NF-kappaB and the levels of TNF-alfa and IL-1 in a cascade, resulting in inflammation. Furthermore, cytokines mentioned above lead to activation of Caspase-3 and other apoptotic pathway mediators, which finally cause neuronal death (44).

Attention paid to the role of inflammation, oxidative stress and the immune system in the relationship between neuropsychiatric disorders and neurodegenerative diseases has been growing recently, and a lot of review articles have summarized recent advances on this topic (44, 45). Activation of microglia and an increase in microglial numbers are characteristics of MSA (21). However, microglia activated can be either pro-inflammatory or anti-inflammatory in phenotype-based on specific cytokines they release (46). To detect inflammation in MSA, Rydbirk et al. searched for cytokine levels in the brains of MSA patients compared to normal controls. They found that IL-2 protein levels and GSK3B (involved in neuroinflammatory pathways) and S100B (neurodegenerative marker) mRNA levels are increased in the prefrontal cortex in MSA brains, while IL-13 and G-CSF protein levels are decreased. Besides, MHC class II and CD45 reactivity are increased in MSA brains (47). These results support local rather than systemic inflammation. Animal studies provide more pieces of evidence. In the MSA mouse model, a key enzyme involved in the production of ROS called myeloperoxidase (MPO) inhibition reduces motor impairment and rescues vulnerable neurons in the striatum, substantia nigra pars compacta, cerebellar cortex, pontine nuclei, and inferior olives. Moreover, MPO inhibition suppresses microglial activation, consistent with the idea that microglial activation might cause a neuronal loss in many brain regions through an inflammatory pathway (48).

### Neurotrophic factors

Besides cytokines relating to inflammation change levels in MSA patients, neurotrophin levels were also measured according to the study mentioned above (47). They measured 5 neurotrophins, brain-derived neurotrophic factor (BDNF), glial-derived neurotrophic factor (GDNF), basic fibroblast growth factor (bFGF), platelet derived growth factor-BB (PDGF-BB), and vascular endothelial growth factor (VEGF), in the dorsomedial prefrontal cortex of MSA patients but found no change compared to healthy controls. Previous studies, however, found contradictory results. For example, one study found increased BDNF-containing axons in the basal ganglia of patients with MSA. These observations suggest that the upregulated expression of BDNF may occur as a protective mechanism in the striatum of MSA patients (48). The discrepancy can be explained by the location of the sample they used. Another study found a decrease of GDNF in the white frontal cortex and to a lesser degree in the cerebellum compared with controls. This result was supported by using transgenic mice expressing human α-Syn in oligodendrocytes as an MSA model (49). Moreover, intracerebroventricular infusion of GDNF improved behavioral performance and ameliorated the neurodegenerative phenotype in this animal model.

## Clinical features of MSA-related depression

### Validated scales for depression

Over the years, several screening tools have been developed for physicians to assess depression. There are over 100 depression rating scales available, which can be observer-report or self-report scales, disease-specific or non–disease-specific scales, subjective scales or objective lab assessments, standard questionnaires, or experience sampling methods (50). This part aims to summarize the use of scales mentioned in the included studies in order to provide an orientating framework for the practicing clinician.

As for observer-report scales, the Hamilton Scale for Depression (HAM-D) has been the most frequently used scale in clinical trials of tricyclic antidepressants since the 1960s (51). The original version consists of 17 items based on the observable behavior during the interview with a score of <8 indicating no depression, a score from 8 to 20 corresponding to mild depression, and a score >20 indicating moderate to severe depression (52). Since the increasing modification of HAM-D, there are 21-, 24-and 29-item versions available for clinical application. The Neuropsychiatric Inventory (NPI) could also be used to estimate the frequency (4-point scale) and severity (3-point scale) of 12 different neuropsychiatric disorders, including depression (53–55).

The Beck Depression Inventory (BDI) is the most widely used self-report scale for determining the severity of depression (56). It is a 21-item multiple-choice scale with excellent psychometric properties in both clinical and general populations. In interpreting BDI scores, a cutoff score of 17 was used to identify moderate to severe depression. The BDI-II is a 1996 revision of the BDI and has also been changed after changing many of the diagnostic criteria for Major Depressive Disorder (57). The Hospital Anxiety and Depression Scale (HADS) was designed for a hospital population, including an anxiety (HADS-A) and a depression (HADS-D) subscale (58). The HADS-D is a 7-item scale and it has been identified as having a cut-off point of 21 for depression. The GDS is another self-report scale (59, 60). The GDS questions are answered simply with a “yes” or “no,” which is suitable for older or demented patients (61).

### The prevalence of depression in MSA patients

A handful of cross-sectional and longitudinal studies have investigated depression in MSA patients, the details are shown in Table 1. Depression is reported to be more frequent in MSA patients than in age- and gender-matched health control (HC) (65, 66, 69, 74, 75, 79). This finding is consistent with the previous conclusion that depression is more common in all disease groups compared to the general population. Evidence has shown that more than 50% of MSA patients had depression and the rate of moderate-to-severe depressive symptoms ranges from 10.4 to 46.3%. Compared to other neuropsychiatric disorders, depression is considered the most important and consistent neuropsychiatric feature in MSA (80).

**Table 1:** Overview of studies reporting clinically relevant depression in MSA.

**References**	**Sample**	**Study design**	**Scale used**	**Prevalence (MSA only)**	**Main findings**
Piol et al. (62)	12 MSA, 12 PD	Observational, cross-sectional	BDI	25% moderate to severe	There was no significant difference in BDI scores between MSA and PD.
Fetoni et al. (63)	12 MSA, 12 PD	Observational, cross-sectional	HAM-D	8.3% dysthymia, 8.3% major depression	PD patients were more depressed than MSA patients.
Benrud-Larson et al. (13)	99 MSA	Observational, retrospective, cross-sectional	BDI	40% mild, 30% moderate to severe, 7% severe	Depression was a strong predictor of QoL and was more prevalent in MSA than in PD; more than 1/3 patients took antidepressant medications.
Tison et al. (64)	50 MSA, 50 PD	Observational, cross-sectional	CES-D	61.2% had depression	Depression was more prevalent in MSA than in PD and greater physical function impairment lead to higher CES-D.
Schrag et al. (4)	115 MSA (72 MSA-P, 43 MSA-C)	Multicenter, observational, cross-sectional	BDI	46.3% moderate to severe	Half of the MSA patients had moderate or severe depression with no significant differences between two subtypes; depression was closely associated with poor HrQoL.
Kawai et al. (65)	35 MSA (21 MSA-C, 14 MSA-P), 21 HC	Observational, cross-sectional	HADS	Not reported	Scores for depression in MSA patients were higher than HC, but no differences between the two subtypes.
Uluduz et al. (66)	16 MSA, 19 PD, 25 HC	Observational, cross-sectional	HAM-D	Not reported	HAM-D test scores were significantly different between the groups, but the worst scores were in the MSA group.
Kao et al. (67)	10 MSA, 8 PD, 12 DLB	Observational, cross-sectional	NPI, GDS	50% had depression	There was a trend for depression to be more frequent in PD and DLB relative to MSA.
Schrag et al. (68)	286 MSA, 188 PSP	Multicenter, observational, cross-sectional	HADS	43% probable depression, 28.1%possible depression	High level of depression in MSA (71.1%) compared to PSP patients (75.4). Depression appeared to be an independent predictor of QoL.
Balas et al. (69)	25 MSA (15 MSA-P, 10, MSA-C), 12 PD, 10 HC	Observational, cross-sectional	BDI	Not reported	Depression could distinguish MSA-P from MSA-C and was related to cognitive decline.
Colosimo et al. (70)	1,130 PD, 34 MSA	Observational, cross-sectional	HAM-D, FAB	Not reported	Depression was mild in MSA patients; the mean HAM-D scores were higher in MSA patients (11.34) than in PD patients (7.91).
Winter et al. (71)	46 MSA (28 MSA-P, 18 MSA-C), 40 PSP	Observational, cross-sectional	BDI	Not reported	Depression is an important independent predictor of low HrQoL. 33 (80.5%) patients received adequate antidepressant therapy.
Siri et al. (72)	61 MSA (39 MSA-P, 22 MSA-C), 20 PD	Multicenter, observational, cross-sectional	GDS, NPI	62% mild to severe	A higher prevalence of depression in MSA (62%) than in PD (10%). Scores of NPI were similar in MSA-C and MSA-P.
Gatto et al. (73)	9 MSA (6 MSA-P, 3 MSA-C)	Observational, cross-sectional	BDI	More than 40% had depression	The presence of depression was observed in more than 40% patients and it tended to be more severely in patients with MSA-C.
Kawahara et al. (74)	33 MSA (8 MSA-P, 25 MSA-C), 106 HC	Cross-sectional	GDS	Not reported	GDS scores higher in MSA-P and MSA-C, than HC and no differences between two subtypes.
Ceponiene et al. (75)	48 MSA, 40 HC	Observational, cross-sectional	NPI, DBI	39.5% mild, 12.5% moderate, 4% severe	The BDI scores correlated positively with the disease duration and negatively with mental QoL. The frequency of depression was higher when assessed by the NPI than by the BDI.
Du et al. (76)	143 MSA (95 MSA-P, 48 MSA-C), 198 PD	Observational, cross-sectional	HAM-D	Not reported	The MSA-P patients showed the highest scores on the HAM-D, followed by MSA-C, and then PD; depression is a strong predictor of poor HrQoL.
Santangelo et al. (77)	44 MSA, 55 PD, 42 PSP	Observational, cross-sectional	BDI-II	50% had depression	The proportion of depressed MSA patients was higher than that of depressed PSP and PD patients.
Zhang et al. (14)	237 MSA (111 MSA-P, 126 MSA-C)	Observational, cross-sectional	HAM-D	38% no depression, 44.3% mild, 17.7% moderate to sever	Depression symptoms are common in MSA patients, especially in female patients and those with longer disease duration, severer disease condition and lower educational level.
Cuoco et al. (78)	55 MSA (29 men, 26 women)	Observational, longitudinal	BDI-II	62.5% had depression	At baseline and follow-up, there was a higher but not significant prevalence of depression in females than in males.
Santangelo et al. (79)	50 MSA (29 MSA-P, 21 MSA-C), 30 HC	Observational, longitudinal	BDI-II	61.5% had depression	Both MSA group had higher scores than HCs on BDI-II but MSA-P and MSA-C had a similar score. After 1 year, the prevalence of depression was consistent in MSA-P and reduced in MSA-C.
Jecmenica-Lukic et al. (80)	47 MSA-P	Cross-section, prospective cohort	NPI, BDI-II, HAM-D	60% had depression	Depression is the most important and consistent neuropsychiatric features in MSA. Patients with a longer disease duration expressed more severe depression. Scale different

MSA, multiple system atrophy; PD, Parkinson's disease; PSP, progressive supranuclear palsy; BDI, Beck Depression inventory; DLB, dementia with Lewy bodies; HAMD, Hamilton Rating Scale Depression; BPRS, brief psychiatric rating scale; MSA-C, multiple system atrophy cerebellar type; MSA-P, multiple system atrophy parkinsonian type; HC, healthy controls; HADS, Hospital Anxiety and Depression Scale; NPI, Neuropsychiatric inventory; GDS, Geriatric Depression Scale; HrQoL, health-related quality of life.

However, those results have been reported without any classification of the disease according to the two motor subtypes. It has been mentioned above that MSA has two subtypes when parkinsonism is predominant, the subtype is referred to as MSA-P; and when cerebellar ataxia is predominant, this subtype is referred to as MSA-C. Since the subtype of MSA-P or MSA-C is determined solely by motor syndrome, it is meaningful to investigate whether each MSA type has its own profile of psychiatric symptoms, especially in terms of depression. Schrag et al. first conducted a multicenter cross-sectional study, whose main purpose was to evaluate the QoL in MAS patients and then investigated 72 MSA-P and 43 MSA-C patients. Using the BDI, they found that half of all MSA patients had moderate to severe depression but the variation between the two groups was not observed (4). Subsequently, Kawai et al. also reported that the prevalence of depression, evaluated by HADS-D, had no significant differences between motor subtypes (65). Similar findings emerged from two subsequent cross-sectional studies, which revealed that MSA-P and MSA-C patients didn't differ in terms of depression (72, 74). However, some other studies showed that the prevalence of depression could distinguish MSA-P from MSA-C. Balas et al. compared depression in 15 MSA-P, 10 MSA-C, 12 PD patients, and 10 HC, and the presence of depression was defined through BDI scores (69). They observed higher BDI scores in MSA-P and PD patients than in HC but found no difference between MSA-C and HC. These results showed that BDI scores were significantly worse in MSA-P than in MSA-C, indicating that MSA-P and MSA-C appear to have a different profile of depression. In addition, Du et al. reported findings of 95 MSA-P and 48 MSA-C patients using HAM-D to compare the presence of depression in two groups, finding that the MSA-P patients had higher scores on HAM-D than the MSA-C patients (76). It seems that depression in patients with MSA-P shows a tendency to be more severe than that of patients with MSA-C, suggesting that the parkinsonian syndrome may contribute more to depressive symptoms than cerebellar ataxia. Collectively, data on whether the prevalence of depression is higher in one of the two subtypes of MSA is controversial. Moreover, a longitudinal study performed a 1-year follow-up investigation of neuropsychiatric profile in 29 MSA-P, 21 MSA-C patients, and 30 matched HC (79). They reported a decreased prevalence of depression over the course of MSA-C and a consistent prevalence in MSA-P. This finding might suggest the hypothesis that the prevalence of MSA-P and MSA-C would change at the different stages. Thus, more prospective, longitudinal data are needed to verify this hypothesis.

As a common type of atypical parkinsonism, MSA shares a common clinical phenomenology with PD, but data on whether they have similar rates of depression is controversial. To compare the occurrence and severity of depressive symptoms between MSA and PD patients, Pilo et al. compared 12 patients with PD and another 12 with MSA using the BDI (62). They found that none of the patients in either group had a major depression episode, but there were three in each group that warranted a clinical diagnosis of moderate depression, suggesting that there may be no difference in depression incidence between MSA and PD patients. In addition, Fetoni et al. reported depressive symptoms in only 2 of the 12 patients with MSA, while 10 of the 12 patients with PD by using the HAM-D, supported by the DSM-IV and BPRS (63). Thus, they came to the conclusion that PD patients are more depressed than MSA patients who, by contrast, may be insensitive to their conditions. However, both these two studies had low reliability due to underpowered sample sizes, necessitating the need for replication with larger studies. For this reason, Benrud-Larson et al. firstly conducted a large sample study (*n* = 99) to document the rate of depressive symptoms in MSA patients by using BDI (13). They found that approximately 80% of the MSA sample reported at least mild depressive symptoms. They contrasted this data against data from a previous study, which reported a lower (19.6%) incidence in PD patients (81). From this, they hypothesized that the rate of depressive symptoms in MSA patients may be considerably higher than that in PD patients. To challenge this finding, Tison et al. conducted a cross-sectional study by comparing the frequency of depression and the severity of self-reported depressive symptoms in 50 MSA compared to 50 sex-, age-, and disease duration-matched PD patients (64). They observed a higher prevalence and severity of depression in MSA (61.2%) than in PD (41%), which is consistent with the hypothesis of Benrud-Larson. Those findings were subsequently confirmed by some observational cross-sectional studies, revealing that MSA patients are more vulnerable to depression and have more depressive symptoms than PD patients (4, 66, 69, 70, 72, 76, 77). Thus, it is reasonable to believe that MSA patients tend to have more difficulties with depression than PD patients, although some small sample size studies held the opposite opinion (62, 63, 67). Progressive supranuclear palsy (PSP) is another common type of atypical parkinsonian syndromes. Few studies have compared the impact of MSA and PSP on psychological conditions. Schrag et al. firstly conducted a large sample size study to directly compare the occurrence of depression evaluated by HADS in 286 MSA and 188 PSP patients (68). The results showed that PSP patients reported significantly more difficulties with depression, indicating that depression was more problematic in PSP than in MSA. Additionally, Santangelo et al. used the BDI to compare psychiatric disturbances in 44 MSA patients, 55 PD, and 42 PSP patients (79). The results showed that the MSA patients had the highest proportion of depression, followed by PSP and then PD, which was not consistent with the conclusion mentioned above, However, there is currently limited literature on the question of whether MSA or PSP patients have a higher prevalence of depression and more research is required.

### The correlation between depression and other clinical characteristics

The progressive clinical course and shortened life expectancy in MSA have major impacts on patients' QoL. Some studies have compared QoL and depression scores in MSA patients over the last few decades to see if depression is an independent predictor of the QoL. Benrud-Larson et al. first applied multiple regression analysis to the investigation of the correlation between depression and QoL scores (13). They conducted a large sample study of 99 MSA patients by using a 100-mm visual analogue scale (VAS) and BDI to assess the QoL and depressive symptoms. The results suggested that depression influenced QoL above and beyond that of disease severity and physical function, indicating a more important role of depression in predicting life quality than disease severity or other debilitating deficits (especially autonomic deficits). However, the use of a single VAS as a measurement may be less reliable than more detailed and informative scales. Thus, other multidimensional scales would be needed to access life quality. In later studies, the Medical Outcomes Study 36-Item Short Form (SF-36) and the EuroQol instrument (EQ-5D) are two widely used measurements of life satisfaction and life quality. Different from the overall QoL evaluated by VAS, those two scales are used to evaluate health-related QoL (HrQoL), which is an important aspect in the healthcare of chronic neurodegenerative diseases (82). The construct of HrQoL is multidimensional and subjective and it includes physical, social, and psychological states (83). Schrag et al. then used EQ-5D to evaluate the HrQoL of 286 MSA patients and performed a multiple linear regression analysis of HrQoL scores and HADS scores. They found that 29% of the variance of the EQ-5D index scores could be independently predicted by the BDI scores (68). It is consistent with the findings from Benrud-Larson et al. that depression is universally an independent and important predictor of poor HrQoL, although Schrag et al. previously reported that depression only contributes to the mental domain of HrQoL but does not appear to be the main factor associated with poor health status (4). Similar to those results, the data of subsequent other HrQoL studies also showed that the presence of depression could reduce life quality significantly without national or geographical differences (71, 76). Collectively, those results emphasize again the importance of depression as a strong and independent predictor of health status for MSA patients and as an important factor for clinical management. Given the limitations of treatment for MSA, great attention should be paid to improving patients' quality of life by providing the treatment for comorbid depression. Moreover, accumulating evidence suggested that a complex interplay between depression and cognitive impairment is established in many neurodegenerative disorders (84, 85). As reported in previous studies, mild to moderate deficits in executive functions, attention and phonological fluency were the most common and prevalent cognitive alterations observed in MSA patients (73, 86, 87). Only one study has applied correlation analysis to the investigation of the association between depression and cognitive outcomes (72). The results showed that depression was frequent in MSA group and it correlated with cognitive abnormalities, involving primarily frontal-executive function, whereas depression didn't account for the differences in cognitive performances between MSA-P and MSA-C. The cognitive symptoms have negative influence not only on patients' QoL, but also on the risk of recurrence of depression. Thus, more attention should be paid to cognitive impairment in MSA patients with depression.

It is also demonstrated that depression in MSA is related to clinical characteristics, such as gender, disease duration, and disease severity. Firstly, some studies have described gender differences in MSA patients. Worse performance in HAM-D scales has been previously reported in MSA females, suggesting that depression occurs more frequently in females than in males (14). In addition, Cucco et al. conducted a longitudinal study of 55 MSA patients to explore gender differences in MSA regarding depressive symptoms evaluated by BDI (78). At baseline and 1-year follow-up, women with MSA had a higher prevalence of depression than men, but the differences were not significant, possibly due to the small sample size. Moreover, accumulating evidence also revealed that in the healthy population and patients with chronic diseases, the female gender was one of the important determinants for depression (88–91). Depression shows gender specificity in which women have a higher lifetime incidence of depression than men (92, 93). Differences in hormone status between men and women may be an explanation for the differences in the occurrence and progression of depression (94–96). Therefore, more attention should be paid to psychological therapy when we manage female MSA patients. Aside from gender differences, Zhang et al. also reported that using the binary logistic regression model, longer disease duration and severer disease conditions were the potential determinants of depression severity in MSA patients (14). Later studies on MSA patients also found similar results with the finding that MSA patients with longer disease duration and severer disease conditions tended to have a worse psychiatric status, especially regarding depression (75, 80). Prior studies have also reported those two characteristics as potential risk factors contributing to the increased severity of depression observed among patients with other neurodegenerative diseases (97). To summarize, it is reasonable to propose that MSA patients, especially female patients and those with longer disease duration and severer disease conditions may be more likely to suffer from depression.

## Neuroimaging findings of MSA-related depression

Advanced neuroimaging techniques have been increasingly applied to investigate the neural substrates of psychiatric disturbances in some neurodegenerative diseases, such as PD, multiple sclerosis (MS), and stroke (98–100). To explain depression with diseases involving the basal ganglia, Mayberg et al. hypothesized that disruption of paralimbic pathways may contribute to both primary depression and depression associated with basal ganglia disease. This network includes nodes such as the frontal cortex, temporal cortex, and striatum (101). A pilot study aiming to test the hypothesis measured cerebral glucose metabolism using positron emission tomography (PET) (102). They found a metabolic decrease in bilateral frontal, parietal and cerebellar cortex and in the left putamen for MSA patients compared to healthy controls, and depression severity was significantly associated with dorsolateral prefrontal glucose metabolism in MSA patients.

Aside from PET, magnetic resonance imaging (MRI) is a useful method for investigating potential changes in the brain and unraveling neural substrates linked to abnormal neuropsychiatric diseases. MRI techniques, mainly including structural MRI and fMRI, allow changes in brain structure and activity to be identified. Accumulating evidence has revealed that the amygdala (AMY), an important structure of the limbic system, plays a pivotal role in delivering and organizing emotional regulation among depression patients (103). Pathologically, this may be due to the aggregation of GCIs, which consist mostly of α-syn, within the AMY (104). Previous studies of MSA patients have detected decreased activation and atrophy in the AMY, suggesting that affective symptoms observed in MSA patients may be attributed to abnormal function and volume of the AMY (105, 106). Recently, a quantitative meta-analysis of voxel-based morphometry (VBM) studies revealed that significant atrophy of AMY has been identified in MSA-P rather than MSA-C subtype, which may be admissible as evidence that severer depressive symptoms occur in the MSA-P subtype (107). Furthermore, in some resting-state functional connectivity (rs-FC) studies, the AMY has been observed to have rich connections with several brain regions, such as the medial prefrontal cortex, insula, thalamus, hippocampus, and striatum (108, 109). Previous research found that in people with depression, the AMY has hypo-connectivity to the dorsolateral prefrontal cortex and anterior insula and hyper-connectivity with the inferior frontal gyrus in individuals with depression (110, 111). Additionally, there is a perception that the increased FC between the ipsilateral AMY and insula is associated with greater alleviation of depressive symptoms over time (112). Thus, it is reasonable to assume that abnormal alteration of AMY-induced FC may be involved in the occurrence of depression. To test this idea, Zhao et al. selected the bilateral AMY as a seed region to compare the FC of the AMY between MSA patients with depression (MSA-D) and MSA patients without depression (MSA-ND) (113). The results identified disrupted amygdala-cortical FC in MSA-D, including the increase of bilateral AMY FC with the left middle frontal gyrus (MFG) while the decreasing of right AMY FC with the left middle occipital gyrus (MOG). Those findings were in line with other studies identifying the roles of MFG and MOG in the pathophysiology of depression (114–116). Furthermore, previous research has found that depressed people have also reported a higher FC of the MFG (117, 118). Although abnormal activation of MOG was observed in depression, no associations were found between depressive symptoms and FC values in MOG (118, 119). The occipital gyrus encompasses visual cortical areas, which could transfer external stimulation to emotion-related cortices (120). It could be speculated that depressive symptoms in MSA patients may be related to specific visual abnormalities, which requires further studies for possible confirmation (113). Recently, an fMRI study showed an increase in the ALFF level of the right middle temporal gyrus (MTG) and it was correlated negatively with depression scores (evaluated by the HAM-D scale) in MSA-D, suggesting that MTG may be an important biomarker of depression in MSA patients. Consistently, accumulating evidence showed that dysfunction within the temporal lobe, where the limbic system is located, was related to depressive symptoms (109, 121–124). In addition, a possible explanation for the negative correlation between ALFF level and depression scores may be that the compensatory up-regulation of MTG was predominated in the early phase of depression because the MSA patients in this study mainly suffered from mild depression. However, this postulate needs verification through further experiments.

## Discussion

MSA is a neurodegenerative disorder causing severe disability and decreased life expectancy. Neuropsychiatric symptoms are very frequent in MSA patients and are dominated by depression. To the best of our knowledge, there has been no up-to-date review regarding depression in MSA patients. To cover this gap, we present the neuropathology, clinical features, and neuroimaging of MSA-related depression in the presence of paper. The exact mechanism of depression in MSA remains to be elucidated and the incomplete understanding of the underlying pathophysiology makes MSA a complex and devastating disease. While an extensive amount of the literature is available for PD, little is known about the nature and clinical correlates of depression in MSA. In this review, we synthesized the corresponding literature and found that neural transmitter dysfunction, including DA, NE, 5-HT, and acetylcholine may be a common denominator between MSA and depression pathology. And beyond that, the role of oxidative stress and neurotrophic factors in the pathogenesis of MSA-related depression has been well-demonstrated. Although evidence from different aspects collectively suggests a wide range of factors contributing to the depression in MSA, most of this evidence is from autopsy and only gives us a correlation between the depression and MSA, calling for more mechanistic studies in the future (125). Furthermore, some factors known or unknown may also play roles in MSA-related depression but the key mediator remains elusive. One direction is to use animal models, such as transgenic mice to get a more mechanistic understanding of the pathology (33, 126, 127). Moreover, the relationship between the gut and the brain is receiving increasing attention these days, with several studies focusing on the change in gut microbiota change in MSA patients (128–130). These changes may influence a lot of factors in the brain, such as inflammatory conditions in the event that microbiota is related to inflammation (129).

Results from a handful of cross-sectional and longitudinal studies showed that more than 50% of MSA patients suffered from depression. In addition, ~10.4–46.3% of MSA patients display moderate-to-severe depressive symptoms.Scores on scales measuring depressive symptoms in MSA patients were significantly higher than in HC, indicating that MSA patients may be more vulnerable to depression than HC. However, disagreement exists regarding the impact of different motor subtypes on the prevalence and severity of depression. Some studies proposed that the prevalence of depression did not significantly differ between MSA-P and MSA-C, while others provided conflicting results, suggesting that MSA-P had a higher level of depression than MSA-C. Even more, a small sample size study (only 6 MSA-P and 3 MSA-C) has reported that depression tended to be more severe in MSA-C patients (73). Given the current disagreement, additional research will be needed to reconcile these discrepancies. Although MSA and PD share a common clinical phenomenology, the prevalence of a clinically relevant rate of depression in MSA patients was significantly higher than that in PD patients. Moreover, both patients with MSA and PSP tend to have more difficulties with depression than PD but whether PSP patients have more severe depression than patients with MSA needs further investigation.

However, it should be noted that the literature on the prevalence of depression in MSA is variable, with studies using different scales for diagnosing depression. For example, Siri et al. used NPI in a multicenter study to compare the prevalence of depression in 61 MSA patients and 20 PD patients and found no statistical differences between the two groups, while they used the GDS to assess depression and showed that the prevalence of depression was significantly higher in MSA (62%) than in PD patients (10%) (72). Considering that NPI and GDS belong to observer-report scales and self-report scales, respectively, it could be speculated that those two kinds of scales measure different aspects of depression. Observer-report scales appear to be associated with external behavioral manifestations while Self-report scales tend to reflect inner emotions. This finding can be explained by the fact that MSA patients themselves appeared to report more depressive symptoms than their caregivers, which is in line with a previous study of PD patients (131). Conversely, another study observed a higher rate of depression in MSA in observers' report (NPI scale) compared with patients' report (BDI scale) (75). This finding can be interpreted as a phenomenon that patients themselves may tend to conceal their emotional state. Moreover, Jecmenica-Lukic et al. tested a set of depression scales in 47 MSA-P patients and found that the overall proportions of depression measured by HAM-D, BDI-II, and NPI were significantly different (80). In conclusion, different scales of depressive symptoms in MSA differed significantly, which makes the interpretation of these results challenging and comparison between different studies difficult or impossible. It should be taken into account when choosing scales to use to measure depression and other neuropsychiatric disorders, as well as when comparing the results of different studies (68).

As an important and independent determinant of HrQoL, depression is an absolute problem that needs to be recognized and solved appropriately. Thus, adequate treatment of depression may be beneficial for improving life satisfaction and life quality, despite the presence of other functional impairments. However, we didn't present the aspect of the treatment of MSA-related depression in this review due to the lack of large-scale randomized controlled trial (RCT) studies to guide the correct therapy for depression in MSA patients. Current therapeutic interventions for MSA patients are only the symptomatic treatments, which mainly target parkinsonism and autonomic failure rather than neuropsychiatric symptoms. Although the evidence to guide treatment is insufficient, receiving pharmacological treatment is necessary for MSA patients who suffer from depression. As for antidepressant medicine, tricyclic antidepressants (TCAs) and selective serotonin reuptake inhibitors (SSRIs) are proven to be effective in PD patients (132, 133). Whether these medicine in MSA patients would take effect remains elusive. In addition, evidence showed that in PD patients, certain symptoms of depression improved with dopaminergic drugs, indicating a role of dopamine in depression (134). However, this may not be suitable for MSA patients, who show a poor response to dopaminergic drugs because of trans-synaptic degeneration of the striatum as a consequence of nigral disease. Similarly, Benrud-Larson et al. reported that BDI scores also did not differ significantly between those taking anti-parkinsonian medications and those not (13). Apart from pharmacological treatment, multiple non-pharmacological approaches, such as cognitive behavioral therapy (CBT) and transcranial magnetic stimulation (TMS) show promising evidence of efficacy in the treatment of depression in some neurodegenerative diseases (135, 136). Further clinical trials should be conducted to investigate whether pharmacological and non-pharmacological interventions may provide an effective means of treating depression or neuropsychiatric disturbances in MSA. In addition, the correlation between depression and clinical characteristics has also been analyzed. Evidence suggested that physicians should pay attention to possible signs and symptoms of depression in MSA patients, especially in female patients and those with longer disease duration and severe disease conditions. In addition, one previous study reported that MSA patients with a lower educational level would express more severe depression (14). Studies on PD patients also found that depressed patients were more likely to have a lower education level (137). However, more studies are needed to confirm this relationship.

A previous systemic review revealed that the implicated brain regions related to depression in PD patients involve a wide spectrum of brain regions, including increased neural activity in the prefrontal regions and decreased functional connectivity between the prefrontal-limbic networks (138). As an atypical parkinsonian syndrome, MSA is thought to have more extensive brain structure abnormalities than PD (107). Only a few studies have investigated neuroimaging evidence of depression in MSA. Evidences have demonstrated the associations between depressive symptoms and cortical regions, such as the frontal lobe and temporal lobe. Besides, the possibility of significant roles of the amygdala in depression, especially abnormal FC of AMY-MFG and AMY-MOG circuits, is also reported. However, the current evidence is far from enough to elucidate the neuroimaging marker of MSA-related depression. More neuroimaging studies should be conducted to fill this knowledge gap and more sophisticated techniques should be applied to yield more concrete results. For example, Neuromelanin-sensitive MRI (NM-MRI) is an advanced technique that aims to detect the content of neuromelanin and visualize neuromelanin-related contrast in the dopaminergic neurons (139). The measurement of neuromelanin loss by using NM-MRI can accurately reflect the progression of the disease over time (140). Previous studies have reported that the application of NM-MRI is useful in the differential diagnosis of parkinsonian diseases, such as PD, MSA, and PSP (141–144). Based on this, Matsuura et al. reported pigmented cell loss in the SNc and LC in MSA patients (35). Given the involvement of the nigrostriatal pathway in mood disturbances, pigmented cell loss observed by NM-MRI can be considered as a potential biomarker to estimate MSA progression and discriminate MSA-D from MSA-ND. Therefore, we have reasons to believe that the application of NM-MRI may provide novel insight into the underlying neural mechanisms of MSA-related depression. Besides, some other well-developed techniques, such as diffusion tensor imaging (DTI), magnetic sensitivity weighted imaging (SWI), arterial Spin Labeling (ASL), quantitative sensitivity magnetization (QSM), and magnetic resonance spectroscopy (MRS) can also be incorporated into future studies.

## Conclusion

In some cross-sectional and longitudinal studies of depression in MSA to date, more than 50% of MSA patients demonstrate significant levels of depression, and this estimate is noted as being higher than current estimates of depression in age-matched HC. As an atypical parkinsonian disease, MSA patients also show more frequent and greater depressive problems than PD patients. Additionally, the difference in depression between the two MSA subtypes has also been reported but the conclusion still remains controversial. Different frequencies of depression among different studies may be explained in part by different assessment scales. Although motor and autonomic dysfunction are the common contributors to low life quality, depression also contributes to a significant degree. Moreover, the proportion of MSA patients, especially female patients, with longer disease duration and severer disease conditions was significantly increased with the increasing severity of depression. Given those observations, it is apparent that screening for depression should be part of routine clinical assessment when working with MSA patients and more attention should be paid to improving patients' life quality through symptomatic therapy for depression. Although depression is one of the common mood disturbances in patients with MSA, its pathophysiology is unclear. There is some evidence from the autopsy that neural transmitter dysfunction, oxidative stress, and neurotrophic factors may be common denominators between MSA and depression pathology. In addition, neuroimaging studies suggest that abnormal alteration of the AMY-MFG and AMY-MOG circuits may play important roles in the progression of depression in MSA. There is also a hypothesized association between the temporal lobe and MSA-related depression. These hypotheses call for more pathological and neuroimaging studies, which could provide new pathophysiological insights. Understanding the underlying mechanisms of MSA-related depression, as well as developing new therapeutic and diagnostic strategies to treat depression will be important in the future.

## Author contributions

QL conceived and wrote the review article. YP wrote the neuropathology section and drew the figure. XC, JWe, and WW independently conducted the search, screened the titles, abstracts, and full texts of the papers. HZ and JWa revised the article. SL, YZ, BY, and DM analyzed the data and drew the table. ZZ and DR gave final approval for publication. All authors contributed to the article and approved the submitted version.

## Funding

Fundamental Research Funds for the Central Universities: No. 2020-JYB-XJSJJ-043.

## Conflict of interest

The authors declare that the research was conducted in the absence of any commercial or financial relationships that could be construed as a potential conflict of interest.

## Publisher's note

All claims expressed in this article are solely those of the authors and do not necessarily represent those of their affiliated organizations, or those of the publisher, the editors and the reviewers. Any product that may be evaluated in this article, or claim that may be made by its manufacturer, is not guaranteed or endorsed by the publisher.
